# Diagnostic and therapeutic impact of cardiac MRI in patients with cryptogenic ischemic stroke

**DOI:** 10.1186/1532-429X-11-S1-P163

**Published:** 2009-01-28

**Authors:** John J Sheehan, George Lin, Jim Connors, Mark Alberts, Karin Dill, Reed Omary, Richard Bernstein, James Carr

**Affiliations:** Northwestern Memorial Hospital, 251 E. Huron Street, Chicago, IL 60611 USA

**Keywords:** Atrial Appendage, Additional Finding, Cryptogenic Stroke, Cardioembolic Stroke, Therapeutic Impact

## Introduction

The aim of our study was to compare cardiac MR (CMR) and Echocardiography (TTE and TEE) used in the detection of intracardiac thrombi in patients with suspected cardioembolic stroke (CES). Over the years, cardiac imaging with different types of sequences has emerged as a non invasive alternative for the detection and chracterisation of intracardiac masses [1–3]. This study examined the utility of CMR for the detection of non-thrombotic additional findings. We will demonstrate the diagnostic and therapeutic impact of CMR.

## Methods

Over a 12 month period between September 2005 and September 2006, 106 consecutive patients with a suspected CES had CMR for the detection of intracardiac thrombi. All CMR examinations were performed on a 1.5 T MR scanner using CINE trueFISP, contrast enhanced MR angiography, delayed enhanced inversion recovery trueFISP and first pass imaging. The clinical information and study reports of echocardiography, CMR, MR Brain and Carotids was retrospectively reviewed.

## Results & discussion

**Figure 1:**
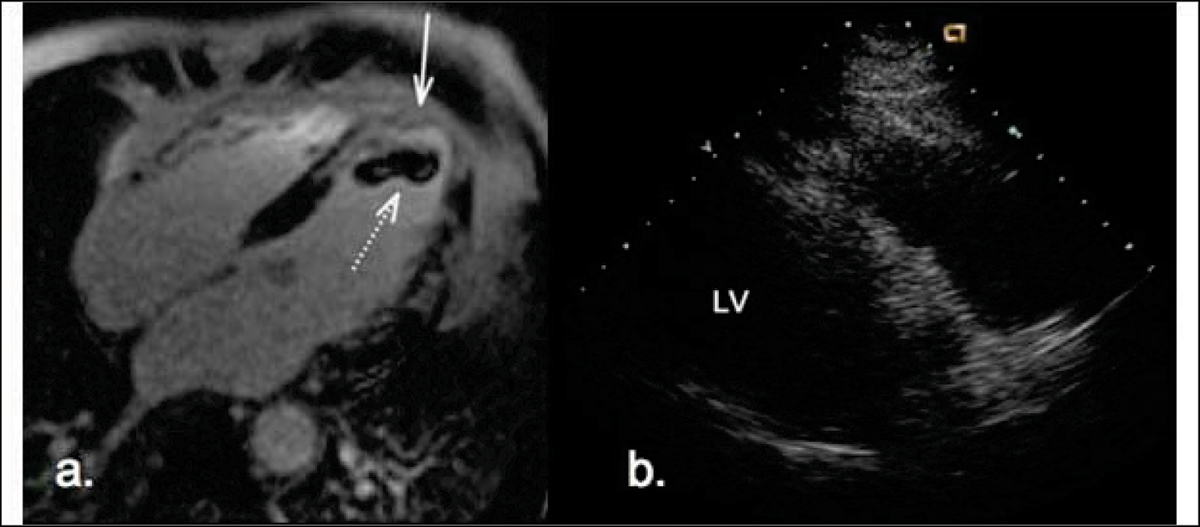
**a. On PSIR Turbo FLASH there is a thrombus (dashed arrow) within the apical region of a mildly dilated left ventricle**. This is located adjacent to a transmural infarct (arrow) b. No thrombus is identified on echocardiography.

93 patients had CMR for suspected CES, revealing 9 thrombi in n = 9 (9.7%) patients. The thrombi were located in the LAA (n = 3), left ventricle (n = 4) and right atrial appendage (n = 3). Of these 9 patients echocardiography was positive in n = 2 (22%), indeterminate in n = 2 (22%) and negative in n = 5 (56%) (Figure 1). No thombi were detected echocargraphy that were not seen on CMR. CMR reported 103 non thrombotic additional findings in n = 53 (57%) of patients compared to echocardiography. Sixty of these were considered significant in n = 38 (40.9%) of patients. Additional findings associated with thrombus formation (acute infarction, scarring and LV aneurysms) were n = 19 (20%) for CMR and n = 7 (7%) for echocardiography. In the n = 9 patients with positive CMR and either false negative (n = 5) or indeterminate (n = 2) echocardiography, secondary preventive therapy changed from antiplatlet agents to anticoagulants (warfarin or heparin) in n = 4 (44%). Presumed stroke mechanism, changed in 3 out of 9 patients (33%), from "cryptogenic" to "cardioembolic". See also Table 1.

**Table 1:** Comparison of MR and echocardiography for thrombus positive studies with thrombus negative studies.

Modality	Number	%
MR & Echo	93	
MR+	9	9.6
Echo-	5	75
Echo+	2	2.1
MR-	2	0

*Two of the negative MRs were suboptimal studies due to motion and prothetic valve artifacts.

## Conclusion

CMR is a non invasive method to detect intracardiac thrombi in stroke patients in whom echocardiograms are either negative or indeterminate. CMR identified intraventricular thrombi in a significant percentage of patients with cryptogenic strokes, leading to a change in secondary preventive therapy. These are complementary studies and when combined maximize the detection of the causes of cardioembolic stroke, may improve the effectiveness of secondary preventive medications, and may detect significant cardiovascular abnormalities that could be missed when only echocardiography is used.
